# Bottom Sediments Reveal Inter-Annual Variability of Interaction between the Ob and Yenisei Plumes in the Kara Sea

**DOI:** 10.1038/s41598-019-55242-3

**Published:** 2019-12-09

**Authors:** A. A. Osadchiev, En. E. Asadulin, A. Yu. Miroshnikov, I. B. Zavialov, E. O. Dubinina, P. A. Belyakova

**Affiliations:** 1 Shirshov Institute of Oceanology, Russian Academy of Sciences, Moscow, Russia; 2 Institute of Geology of Ore Deposits, Petrography, Mineralogy and Geochemistry, Russian Academy of Sciences, Moscow, Russia; 3 Water Problems Institute, Russian Academy of Sciences, Moscow, Russia

**Keywords:** Element cycles, Physical oceanography

## Abstract

River discharge is the main source of terrigenous sediments in many coastal areas adjacent to estuaries and deltas of large rivers. Spreading and mixing dynamics of river plumes governs transport of suspended sediments and their deposition at sea bottom at these areas. Generally river plumes have very large synoptic and seasonal variability, which cannot be reconstructed from structure of bottom sediments due to their small accumulation velocity. However, bottom sediments can be indicative of variability of river plumes on inter-annual and decadal time scales. In this study we focus on the large Ob and Yenisei buoyant plumes formed in the central part of the Kara Sea. These plumes interact and mix in the area adjacent to the closely located Ob and Yenisei gulfs. Suspended sediments carried by these river plumes have significantly different geochemical characteristics that can be used to detect Ob or Yenisei origin of bottom sediments. Using new geochemical methods we revealed dependence between spreading patterns of these plumes and spatial distribution and vertical structure of bottom sediments in the study area. This relation is confirmed by a good agreement between local wind and discharge conditions reconstructed for 1948–2001 and vertical structure of bottom sediments.

## Introduction

**Figure 1:**
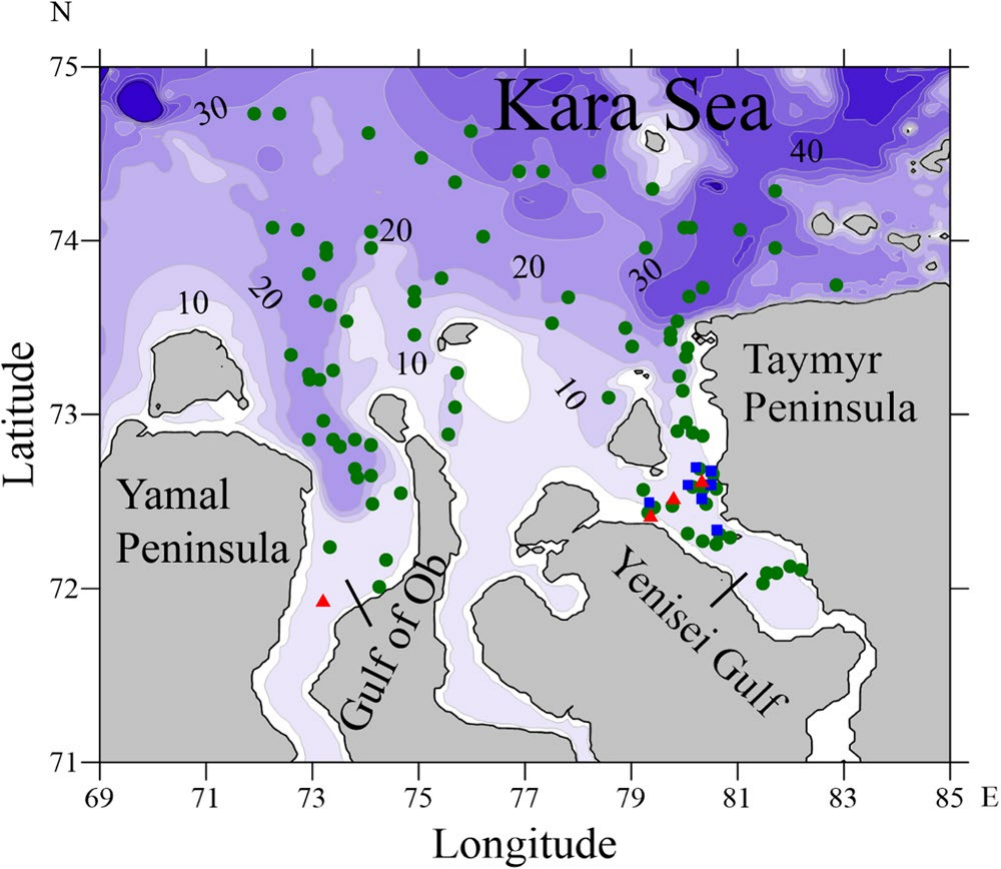
Bathymetry of the central part of the Kara Sea, locations of the sediment samples of seafloor surface layer (green circles), the bottom sediment core samples (blue squares), and the water samples of freshened sea surface layer (red triangles) (**a**).

The Yenisei and Ob rivers are the first and the third largest Arctic rivers, their basin areas are 2580000 and 2990000 km^2^. Annual freshwater discharge from the Gulf of Ob (485 km^3^) and the Yenisei Gulf (630 km^3^) to the Kara Sea provides about a third of the continental runoff to the Arctic Ocean^1–3^. The Ob and Yenisei gulfs are closely located; the distance between their mouths is approximately 150 km (Fig. 1). As a result, the Ob and Yenisei discharges form large river plumes that collide and coalescence in the area adjacent to the Ob and Yenisei gulfs^4,5^. The processes of interaction and mixing of the Ob and Yenisei plumes governs structure and dynamics of surface layer in the central part of the Kara Sea^4,6–9^ and influences many local physical, biological, and geochemical processes that were addressed in the previous studies^10–15^.

**Figure 2:**
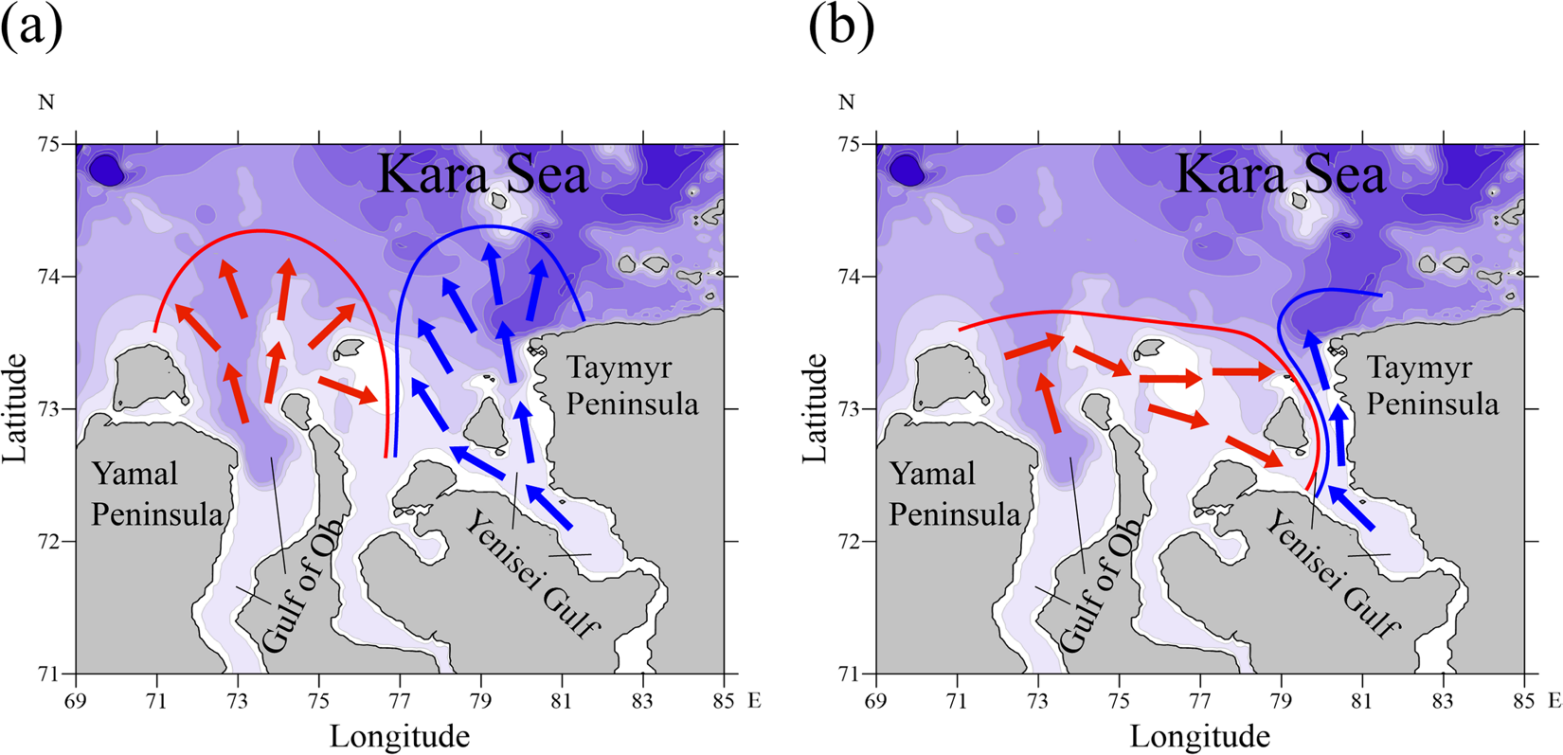
Schematic of the general patterns of the interaction between the Ob and Yenisei plumes: northward spreading and offshore collision of the Ob and Yenisei plumes (**a**), eastward intrusion of the Ob plume into the Yenisei Gulf and isolation of the Yenisei plume (**b**).

Generally, the Ob and Yenisei plumes are spreading northward from the respective gulfs, occupy wide areas, and collide in the open sea between the Yamal and Taymyr peninsulas (Fig. 2a). This circulation scheme was described in several studies based on *in situ* data and numerical modelling^7–9,16^ and is a typical interaction pattern between closely located river plumes^17,18^. However, based on *in situ* and satellite data, *Osadchiev et al*.^5^ revealed another pattern of interaction between the Ob and Yenisei plumes, which is substantially different from that described above. Strong northward wind forcing during the periods of high discharge of the Ob River causes eastward spreading of the Ob plume and its intrusion into the Yenisei Gulf (Fig. 2b). As a result, the Ob plume occupies a large area, while the Yenisei plume is arrested along the Taymyr Peninsula.

In this work we show that bottom sediments in the study area can provide important information about the interaction between the Ob and Yenisei plumes. The analysis of origin of river-borne bottom sediments collected in the study area reveals inter-annual variability of the typical spreading patterns of the Ob and Yenisei plumes. In particular, we detect several layers of mixed Ob-Yenisei origin at the bottom sediment core samples collected in the Yenisei Gulf which are indicative of long-term periods of frequent intrusion of the Ob plume into the Yenisei Gulf described above. Local wind reanalysis data and gauge measurements of the Ob and Yenisei river discharges confirm the dependence between the origin of bottom sediments and the obtained intra-annual variability of the interaction patterns between the Ob and Yenisei plumes.

## Results

Geologically different types of the catchment areas of the Ob River (friable Mezo-Cenozoic deposits of clays, sandstones, marls, siltstones, etc.) and the Yenisei River (basalt, diabase, gabbro, and dolerites) result in distinction of geochemical and mineralogical characteristics of suspended sediments carried by the Ob and Yenisei plumes^10,14^. Concentrations of certain chemical elements from the lithophile group are significantly different in suspended sediments of the Ob and Yenisei rivers^19,20^. Therefore, concentrations of these geochemical tracers in bottom sediments are indicative of their Ob and Yenisei origin.

Concentrations of multiple chemical elements and compounds were determined in 284 and 442 bottom sediment samples collected in the Ob and Yenisei gulfs. Based on the obtained mean values, standard deviations of their concentrations, and correlation analysis, we determined the elements and compounds, which can be regarded as the stable tracers for suspended sediments of the Ob (Rb, Cs, La, Ce, Nd, Sm, Eu, Yb, Lu, Hf, Ta, Th, U) and Yenisei (Sc, Cr, Co, CaO) origins. The detailed description of determination of these trace elements and compounds and the related statistical analysis are provided in^19,20^.

**Figure 3:**
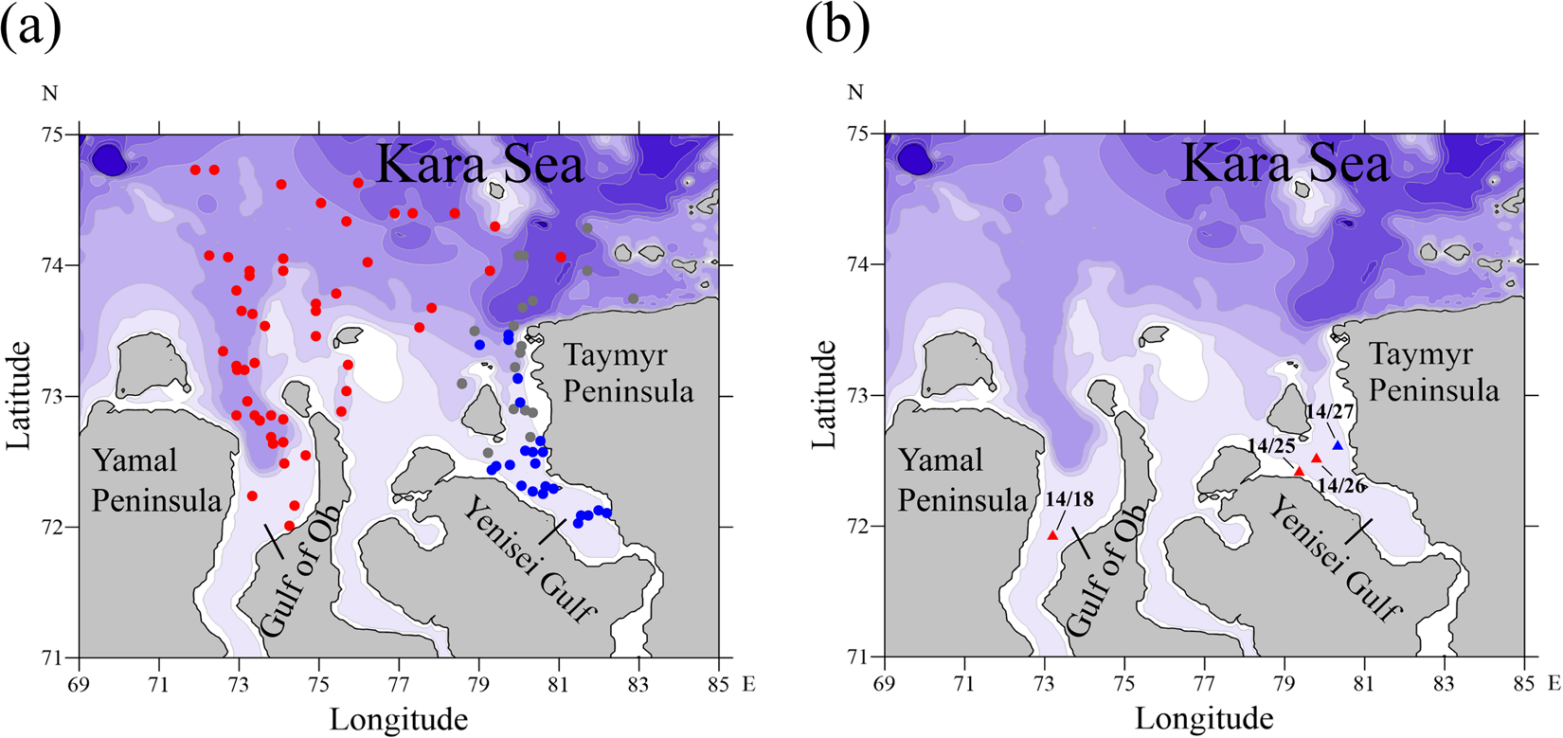
Locations of sediment samples of seafloor surface layer of the Ob (red), Yenisei (blue), and mixed Ob-Yenisei (gray) origin identified by the additive geochemical criterion. (**a**) Locations of water samples of freshened sea surface layer of the Ob (red) and Yenisei (blue) origin on 22 August 2014 identified by the analysis of oxygen and hydrogen isotope compositions (**b**).

We applied the additive geochemical criterion for distinguishing the Ob and Yenisei origin bottom sediments as follows: *AGC*_*O/Y*_ = ln(*E*_*O*_/*E*_*Y*_), where *E*_*O*_ = (*Rb* + *Cs* + *La* + *Ce* + *Nd* + *Sm* + *Eu* + *Yb* + *Lu* + *Hf* + *Ta* + *Th* + *U*)/13 is the sum of normalized concentrations of the trace elements of suspended sediments of the Ob River, *E*_*Y*_ = (*Sc* + *Cr* + *Co* + *CaO*)/4 is the sum of normalized concentrations of the trace elements of suspended sediments of the Yenisei River. Figure 3a shows that the additive geochemical criterion distinctly distinguished the Ob origin of bottom sediments (*AGC*_*O/Y*_ > 0.1) in the Gulf of Ob and the Yenisei origin of sediments (*AGC*_*O/Y*_ < −0.1) in the Yenisei Gulf. However, small absolute values of the additive geochemical criterion (−0.1 < *AGC*_*O/Y*_ < 0.1) registered in many stations in the Yenisei Gulf and adjacent sea areas indicate that the considered samples have mixed Ob-Yenisei origin.

Intrusion of the Ob plume into the northern part of the Yenisei Gulf was also detected by the oxygen and hydrogen isotope compositions of the water samples collected in the Gulf of Ob (station 14/18) and in the Yenisei Gulf (stations 14/25, 14/26, and 14/27) on 22 August 2014 (Fig. 3b). Salinity at station 14/18 was equal to 0 from surface to bottom (14 m deep) indicating that the whole water column was formed by the Ob River water. Values of *δD* and *δ*^18^*О* at this station were equal to −131.4 ± 0.3‰ and −17.6 ± 0.1‰. Values of *δD* and *δ*^18^*О* in the surface layer at stations 14/25 and 14/26 located in the western part of the Yenisei Gulf were similar (−133.4 – −134.6‰ and −17.6 – −17.8‰), while salinity of the 8–10 m deep freshened surface layer was equal to 1–2. On the other hand, the isotopic composition registered in the freshened surface layer in the eastern part of the Yenisei Gulf was significantly different. Values of *δD* and *δ*_18_*О* in the surface layer at station 14/27 were equal to −120.4‰ ± 0.3‰ and −15.7 ± 0.1‰, while salinity of freshened surface layer was 2. Therefore, we presume that during the field measurements the Ob plume was spreading in the western part of the Yenisei Gulf and was registered at stations 14/25 and 14/26, while the Yenisei plume occupied the southern and eastern parts of the gulf.

The additive criterion is less influenced by low-amplitude noise variability of concentrations of trace elements and compounds; however, it is not stable in case of anomalously large concentrations, as compared to the Bayesian criterion. Therefore, in this study we applied the additive criterion to identify origin of the sediment samples of seafloor surface layer, which have relatively large volumes. On the other hand, the analysis of origin of relatively small sections of the sediment cores prone to fluctuations and outliers was performed using the Bayesian criterion that provides more stable results.

**Figure 4:**
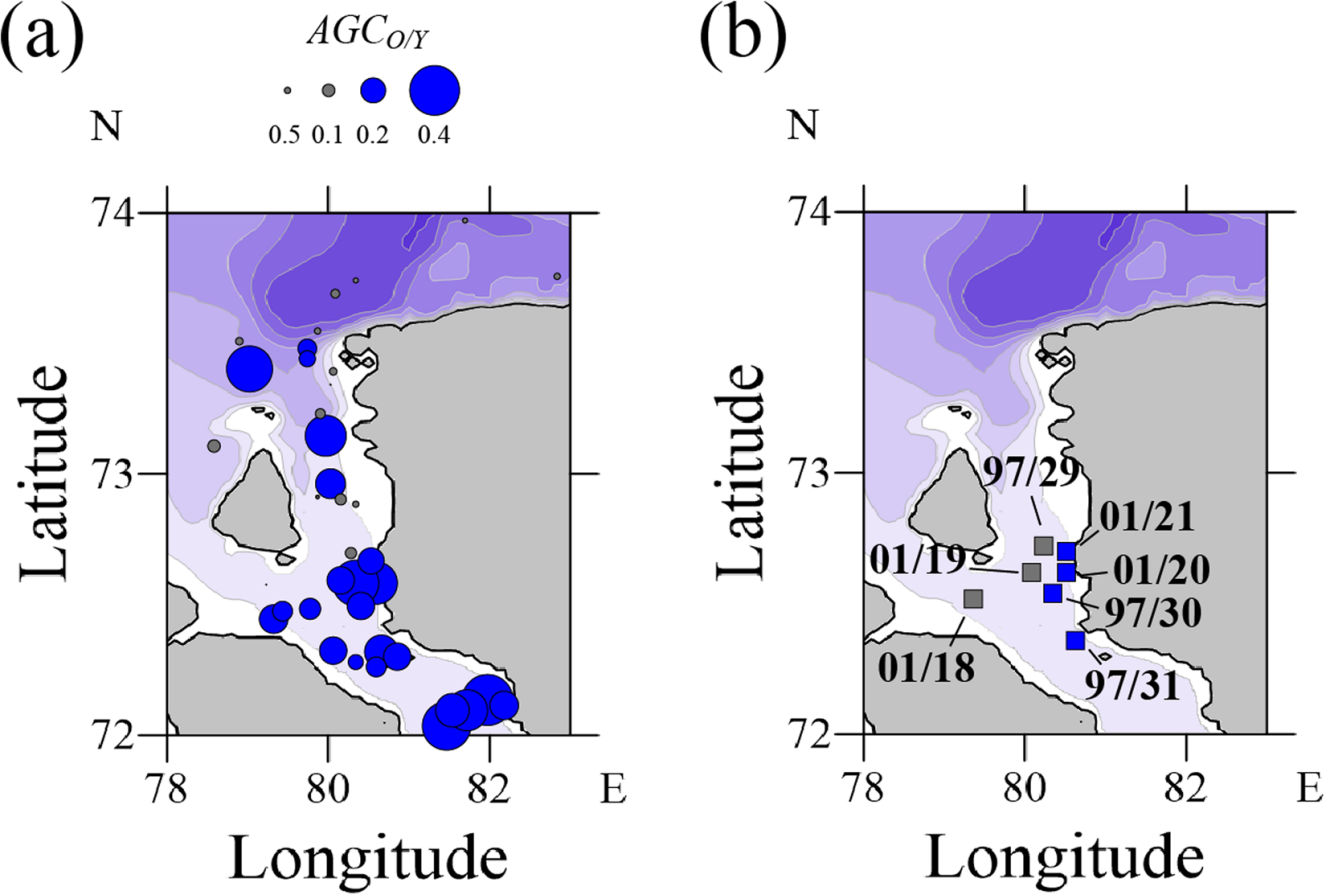
Locations of the sediment samples of seafloor surface layer of the Yenisei (blue circles) and mixed (gray circles) origin in the Yenisei Gulf identified by the additive geochemical criterion (zoomed part of Fig. 3a), sizes of the circles represent absolute values of *AGC*_*O/Y*_. (**a**) Location of the bottom sediment core samples of the Yenisei (blue squares) and mixed (gray squares) origin in the Yenisei Gulf identified by the Bayesian geochemical criterion (**b**).

We analyzed the bottom sediment core samples collected at stations 97/29 (sea depth was 16 m), 97/30 (14 m), 97/31 (13 m) in 1997 and stations 01/18 (13 m), 01/19 (28 m), 01/20 (16 m), 01/21 (16 m) in 2001 located in the southern part of the Yenisei Gulf (Fig. 4b). This area is characterized by extremely rapid sedimentation rate caused by the interaction and mixing between freshwater discharge and saline sea water^14,19–26^. Northward from this area salinity in the surface layer and sediment load sharply decrease^5,22,26^. The lithologic composition of bottom sediments is relatively homogenous for the southern part of the Yenisei Gulf. Bottom sediments are mainly composed of pelitomorphic silt of olive-gray to black color with admixture of aleuritic and psammitic matter^21,23,24,26^. Due to extremely rapid accumulation of river-borne sediments, influence of coastal erosion on the local sediment input is relatively low that results in small share of coarse sediment fraction at the considered stations^22,27^.

**Figure 5:**
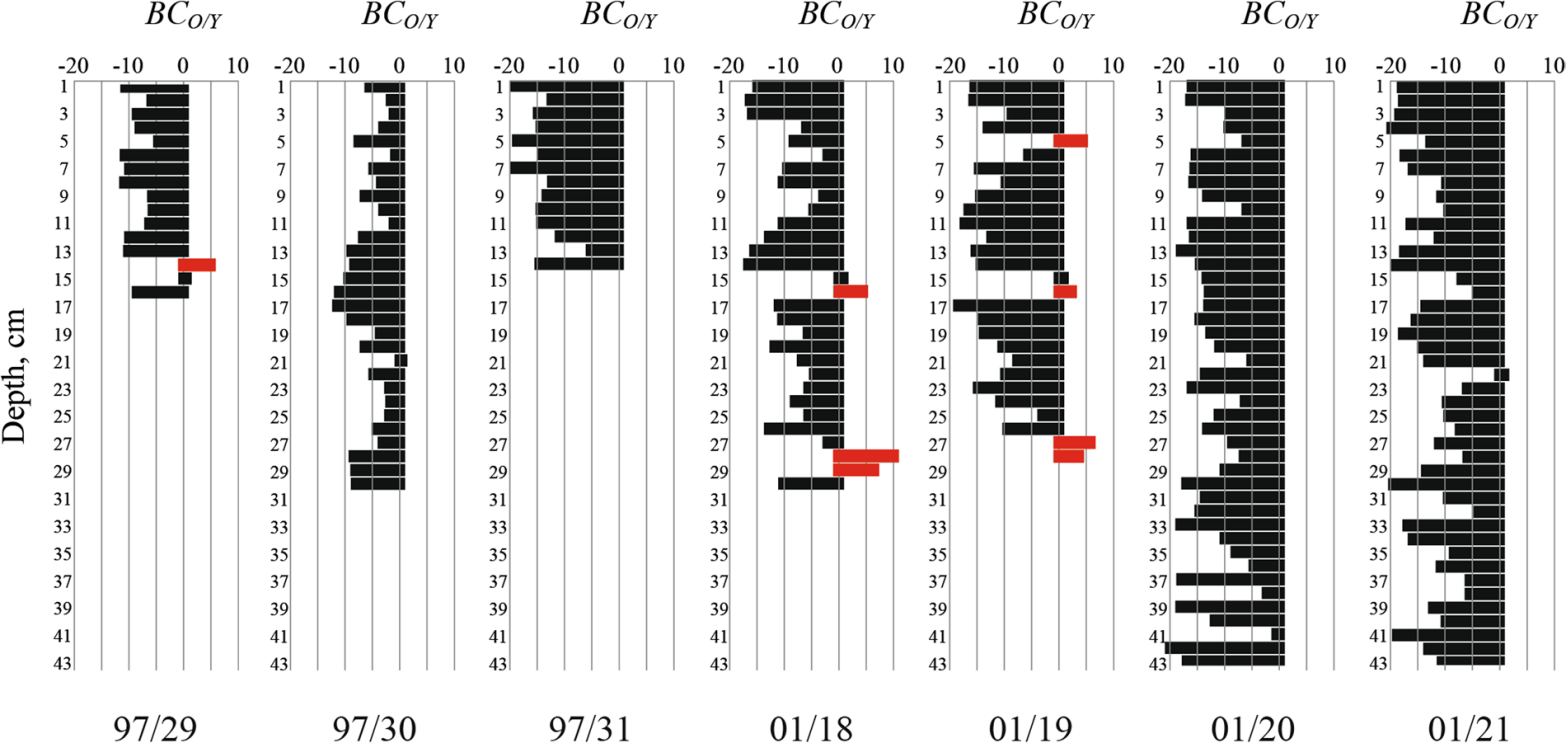
The clustered values of the Bayesian geochemical criterion (*BC*_*O/Y*_) at the 1-cm deep layers of the bottom sediment core samples collected at stations 97/29, 97/30, 97/31, 01/18, 01/19, 01/20, and 01/21. The first cluster contains negative and small positive values of *BC*_*O/Y*_ and indicates bottom sediments of the Yenisei origin (black bars), the second cluster contains large positive values of *BC*_*O/Y*_ and indicates bottom sediments of the mixed Ob-Yenisei origin (red bars).

We applied the Bayesian geochemical criterion for distinguishing the Ob and Yenisei origin bottom sediments in the collected sediment core samples using the same set of the trace elements and compounds, as for the additive geochemical criterion. The resulting distribution of bottom sediments of the Yenisei and mixed Ob-Yenisei origin detected in the Yenisei Gulf by the additive geochemical criterion (Fig. 4a) is consistent with results of analysis of the bottom sediments cores collected in the Yenisei Gulf by the Bayesian geochemical criterion (Fig. 4b). The bottom sediment cores collected at stations 97/30, 97/31, 01/20, and 01/21 located in the southern and eastern parts of the Yenisei Gulf have the distinct Yenisei origin of all extruded core layers. On the other hand, the Bayesian geochemical criterion shows significant inhomogeneity of the vertical structure of the bottom sediments cores collected at stations 01/18, 01/19, and 97/29 (Fig. 5). The majority of the analyzed layers were formed by sediments deposited from the Yenisei plume due to negative value of *BC*_*O/Y*_ at these layers. However, at several layers *BC*_*O/Y*_ > 0, that indicates their mixed Ob-Yenisei origin. In order to highlight these anomalous layers, we clustered vertical levels of the considered sediment cores to two clusters, namely, of the Yenisei origin and of the mixed Ob-Yenisei origin using expectation–maximization algorithm. Depths of these layers are consistent at these stations and are equal to 14–16 and 26–27 cm (Fig. 5). The sediment cores at stations 01/18 and 01/19 were collected in 2001, while the core at station 97/29 was collected in 1997, i.e. six years earlier.

*Osadchiev et al*.^5^ provided the following wind and discharge conditions of the intrusion of the Ob plume into the Yenisei Gulf: *M*_*x*_ > 100 kg m^−1^ s^−1^, *M*_*y*_ < 100 kg m^−1^ s^−1^, *Q*_*O*_ > 20000 m^3^ s^−1^, and *Q*_*Y*_/*Q*_*O*_ < 10, where *M*_*x*_ and *M*_*y*_ are the meridional and zonal Ekman transport averaged over the study area (72.5–75 °N, 72.5–80 °E) and over a period of 3 days, *Q*_*O*_ and *Q*_*Y*_ are the Ob and Yenisei discharge rates. In particular, local wind (*M*_*x*_ = 111 kg m^−1^ s^−1^, *M*_*y*_ = −140 kg m^−1^ s^−1^) and discharge (*Q*_*O*_ = 32300 m^3^ s^−1^, *Q*_*Y = *_17700 m^3^ s^−1^) conditions preceding the period of collecting water samples on 22 August 2014 were favorable for the intrusion of the Ob plume into the Yenisei Gulf that is consistent with the results of the isotope analysis. Based on these inequalities applied to NCEP/NCAR wind reanalysis and river discharge data, we identified periods favorable for intrusion of the Ob plume into the Yenisei Gulf during ice-free seasons of 1948–2016. We obtained that these intrusions were especially frequent in 1948–1949 and 1968–1975. We presume that frequent intrusions during these periods can result in accumulation of large volume of suspended sediments of the Ob origin in the Yenisei Gulf that can be detected at the certain layers of local bottom sediments. However, in order to compare these periods and vertical structure of the sediments cores collected at stations 01/18, 01/19, and 97/29 we need to determine the accumulation velocity of bottom sediments in the Yenisei Gulf.

**Figure 6:**
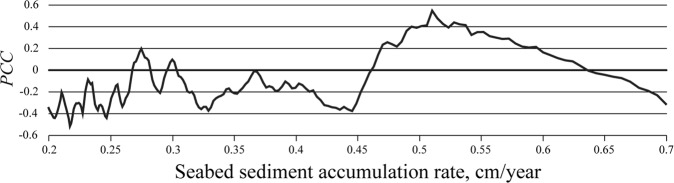
Values of the Pearson correlation coefficient (PCC) between the averaged sum of the Bayesian geochemical criterion (*BC*_*O/Y*_) applied to 1-cm thick layers of the bottom sediment core samples collected at stations 01/18, 01/19, and 97/29, on the one hand, and duration of wind and discharge conditions favorable for the intrusion of the Ob plume into the Yenisei Gulf during 1948–2001 summarized for the sets of periods corresponding to a range of bottom sediment accumulation rates from 0.2 to 0.7 cm/year, on the other hand.

Previous studies revealed that this velocity is estimated, first, as 0.2–0.5 cm/year according to correspondence between the depths of peak values of ^137^Cs and the years of accidental intense radioactive emission to the Yenisei River^23,24^ and, second, as 0.3–0.6 cm/year according to correspondence between the depths of peak values of ^137^Cs at the sediments cores collected at the same place in 1997–1999 and in 2016, i.e., with time lag equal to 17–19 years^25^. Figure 5 illustrates that vertical distribution of *BC*_*O*/*Y*_ is consistent at closely located stations 01/18, 01/19, and 97/29. Therefore, we presume that the long-term average accumulation velocities of bottom sediments at these stations have similar values between 0.2 and 0.6 cm/year. We considered different bottom sediment accumulation velocities in this range and for every individual accumulation velocity we split the period of 1948–2011 into a set of periods that provide 1-cm thick sediment layers. Then we calculated duration of wind and discharge conditions favorable for intrusion of the Ob plume into the Yenisei Gulf during these sets of periods, i.e., number of intrusion days during time periods that provide 1-cm thick sediment layers for different accumulation velocities. Then we calculated the Pearson correlation coefficient (PCC) between the averaged vertical distribution of *BC*_*O*/*Y*_ at stations 01/18, 01/19, and 97/29, on the one hand, and the sets of numbers of intrusion days, on the other hand for different accumulation velocities. The obtained range of maximal values of PCC (0.4–0.5) indicates the best approximation of the long-term average bottom sediment accumulation rate at the considered stations that is equal to 0.49–0.54 cm/year (Fig. 6). Relatively low value of the maximal PCC is caused by intra-annual variability of annual sediment load rates in the Yenisei Gulf due to variability of annual river runoff.

**Figure 7:**
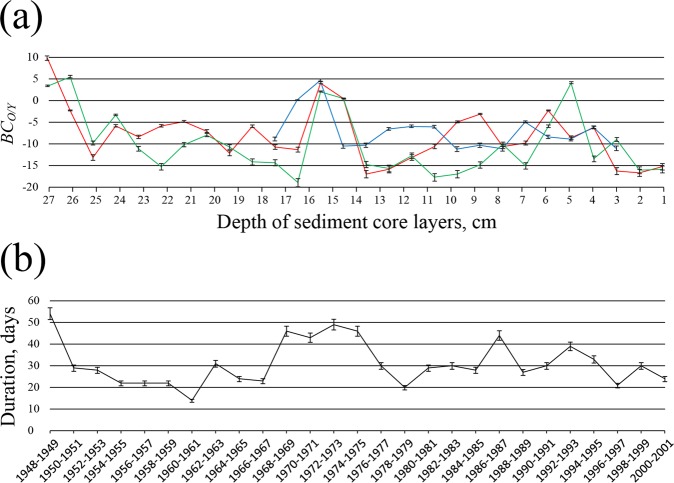
Values of the Bayesian geochemical criterion (*BC*_*O/Y*_) applied to 1-cm thick layers of the bottom sediment core samples collected at stations 01/18 (red line), 01/19 (green line), and 97/29 (blue line). (**a**) Duration of wind and discharge conditions favorable for the intrusion of the Ob plume into the Yenisei Gulf summarized for 2-year long periods during 1948–2001 (black line). (**b**) Error bars represent 95% confidence intervals.

The obtained high values of PCC confirm that the long-term average bottom sediment accumulation rate at the considered stations can be approximated by 0.5 cm/year. Second, they illustrate that the Ob/Yenisei origin of the extruded 1-cm thick layers of the bottom sediment core samples shows a good accordance with duration of the intrusions of the Ob plume into the Yenisei Gulf during the respective 2-year long periods (Fig. 7). In particular, long periods of frequent intrusion of the Ob plume into the Yenisei Gulf (1948–1949 and 1968–1975) resulted in accumulation of large volume of suspended sediments carried by the Ob plume in the Yenisei Gulf. Therefore, values of *BC*_*O/Y*_ at the respective core layers (14–17 and 26–27 cm deep) are equal to 0–10 revealing mixed Ob-Yenisei origin of these layers (Fig. 7). On the other hand, the periods of rare intrusions of the Ob plume into the Yenisei Gulf (1950–1967, 1976–1985, 1988–1991, and 1994–2001) correspond to negative values of *BC*_*O/Y*_ and the distinct Yenisei origin of the respective core layers.

We analyzed frequency and duration of the intrusions of the Ob plume into the Yenisei Gulf during ice-free periods (July – October) in 1948–2016 estimated from wind reanalysis and river discharge data. The majority of intrusions lasted from 1 to 6 days, therefore, this process is governed by atmospheric variability on synoptic time scale. The average total duration of the intrusions during a year is only 16 days, however, it has substantial inter-annual variability from 0 (2004), 1 (1965), and 3 days (1956, 1967) to 30 (2002), 32 (2016), and 35 days (1969) caused mainly by the inter-annual variability of local atmospheric circulation. The longest registered intrusion occurred in September – October 1969 and lasted during 22 days. As a result, frequent and/or long intrusions during certain weeks and months can largely influence physical, biological, and geochemical processes in the Yenisei Gulf.

## Discussion

Spreading dynamics of river plumes and the associated transport of river-borne suspended sediments play a key role in accumulation of bottom sediments in river estuaries and adjacent shelf areas^18,28–30^. Generally river plumes have very large synoptic and seasonal variability^31–34^, which cannot be reconstructed from spatial distribution and vertical structure of bottom sediments due to their small accumulation velocity. However, bottom sediments can be indicative of variability of river plume dynamics on inter-annual and decadal time scales, especially in coastal areas characterized by rapid sedimentation rates. In this study we reveal correlation between the spreading and interaction patterns of the Ob and Yenisei plumes, on the one hand, and the spatial distribution and vertical structure of bottom sediments in the Ob and Yenisei gulfs and the adjacent sea area, on the other hand. This relation is confirmed by a good accordance between the vertical structure of bottom sediments in the Yenisei Gulf and local wind and discharge conditions in 1948–2001.

We show that sedimentation from the Ob plume dominates in the Gulf of Ob and the area located to the north from the Gulf of Ob, while sediments of the Yenisei origin prevail in sea bottom only in the southern part of the Yenisei Gulf. Therefore, despite the fact that annual discharge rates of the Ob and Yenisei rivers are similar and the Ob and Yenisei gulfs are closely located, the transport and deposition patterns of suspended sediments carried by these rivers are significantly different. The observed eastward shift of the Ob-origin bottom sediments (Fig. 3a) is caused by prevailing eastward transport of river plumes formed by the Ob, Yenisei and other large Arctic rivers^35,36^ due to direction of the Coriolis force that plays an important role in their dynamics. On the other hand, eastward shift of the Yenisei-origin bottom sediments is hindered by coastline of the Taymyr Peninsula that results in prevailing northward alongshore spreading of the Yenisei plume and its mixing with the Ob plume. This is supported by location of bottom sediments of the mixed Ob-Yenisei origin to the north and west from the Yenisei Gulf and in the northern part of the Yenisei Gulf indicating the mixing areas between the Ob and Yenisei plumes (Fig. 4a). Also concentration of suspended sediments in the Ob River (38 g/m^3^) is several times greater than in the Yenisei River (10 g/m^3^)^37^ that results in predominant role of sediments carried by the Ob plume in the areas of mixing between the Ob and Yenisei plumes.

Certain wind and discharge conditions cause eastward spreading of the Ob plume and its intrusion into the western part of the Yenisei Gulf ^5^. Formation of these intrusions is governed by synoptic variability of local atmospheric circulation; generally they last during less than 6 days. However, the total annual duration of these intrusions shows significant inter-annual variability from 0 to 35 days in a year. Therefore, this feature is common during ice-free periods in the study region and can largely influence the structure of the freshened surface layer in the Yenisei Gulf, influence spreading and mixing dynamics of the Ob and Yenisei plume in the Kara Sea. The intrusions of the Ob plume into the Yenisei Gulf also strongly affect local sedimentation process. Based on the analysis of the sediment core samples, we reveal that the frequent intrusions of the Ob plume into the Yenisei Gulf in 1948–1949 and 1968–1975 resulted in accumulation of large volume of suspended sediments of the Ob origin in the Yenisei Gulf.

We presume that sediment resuspension caused by wind and tides is not intense at the study region and has low impact on the sedimentary environment due to the following reasons. The majority of storm events in the study area occurs in November – June due to enhanced cyclonic activity, however, during this period the Yenisei Gulf is covered by ice^16^. During ice-free periods storm events are rare and wind-induced turbulence limitedly affects water beneath the deep (5–10 m) and strongly stratified freshened surface layer formed in the Yenisei Gulf^5,12,16,38^. Tidal currents in the Yenisei Gulf are relatively low, their velocities do not exceed 0.2 m/s^39,40^. Analysis of structure and granulometric composition of bottom sediments in the core samples considered in this study did not reveal any evidence of sediment resuspension. In particular, no gradational stratification was detected. As a result, we presume that wind and tidal-induced resuspension of bottom sediments limitedly affects their distribution in the southern part of the Yenisei Gulf. Therefore, the vertical structure of sediment cores collected at stations 01/18, 01/19, and 97/29 reflects the dynamics of the Yenisei and Ob plumes.

This result is also supported by similar vertical structure of the sediment cores collected at stations 01/18, 01/19, and 97/29. The layers of the mixed Ob-Yenisei origin were detected by the geochemical analysis at the same depths (14–17 and 26–27 cm) at three different stations located on a distance of more than 25 kilometers. If sedimentation environment in the study area was significantly affected by resuspension, spatial variation of *BC*_*O/Y*_ will be large and no distinct accordance will be observed between depths of the layers of the mixed Ob-Yenisei origin at these stations. Vertical variation of *BC*_*O/Y*_ at the considered core samples also supports our assumption that resuspension of bottom sediments limitedly affects their distribution in the study area. Vertical profile of *BC*_*O/Y*_ is characterized by similar positive values at the majority of the layers indicating their Yenisei origin with several distinct “outliers”, i.e., layers of the mixed Ob-Yenisei origin. If sedimentation environment in the study area was significantly affected by resuspension, transitions between the neighboring layers of different origin will be smooth and no jumps of *BC*_*O/Y*_ value will be observed.

Finally, we show that the additive and Bayesian geochemical criteria are effective tools for analysis of origin of bottom sediments. Distinguishing sediments of the Ob and Yenisei origin is important for study processes of spreading, mixing, and settling of river-borne matter and many related issues, in particular, identification of sources of radioactive pollution that is accumulated in bottom sediments in the study area^14^. Moreover, interaction between buoyant plumes formed by discharges of neighboring rivers is a common feature in many other world coastal areas. Therefore, bottom sediments can be indicative of these interactions, as was described for the interaction between the Ob and Yenisei plumes. However, usage of geochemical criteria has several important limitations. First, suspended sediments of the considered rivers have to have distinct differences of geochemical characteristics. However, rivers that discharge to sea in close proximity, especially small ones, commonly have adjacent watershed basins with similar geochemical and mineralogical properties. Second, this method requires intense accumulation of river-borne sediments in coastal area that results in formation of detectable layers of bottom sediments of certain origin at seafloor. Finally, bottom resuspension can strongly modify structure of bottom sediments and hinder distinguishing sediments of different origin. Therefore, the method described in this study is generally applicable for medium-size and large rivers with intense sediment accumulation rates and low resuspension in coastal area adjacent to their mouths. In these cases the additive and Bayesian geochemical criteria hold promise of providing improved qualitative and quantitative insights into processes of transport and accumulation of river-borne suspended sediments in many world coastal regions.

### Data and methods

#### Data used

The *in situ* data used in this study were collected during seven oceanographic field surveys in the Kara Sea including the 22^th^, 28^th^, 32^nd^, 35^th^ and 36^th^ cruises of the R/V “Akademik Boris Petrov” in 1995–2001, the 128^th^ cruise of the R/V “Professor Shtokman” in 2014, and the 66^th^ cruise of the R/V “Akademik Mstislav Keldysh” in 2015 (Fig. 1a). The *in situ* data included sediment samples of seafloor surface layer, bottom sediment core samples, and water samples of the freshened sea surface layer (Fig. 1a).

The seafloor surface samples were collected by grab samplers, while the core samples were collected by a box corer. The sediment cores were sectioned at 1 cm intervals, each extruded section was sliced off the core using a kapron line and a plastic knife, a 0.5-cm thick outer part of the core was removed. Then samples were dried in a desiccator at 60 °C^14^. The instrumental neutron activation analysis was used to determine concentrations of the stable elements and their compounds in the samples of bottom sediments including the following, which were used in this study: Rb, Cs, La, Ce, Nd, Sm, Eu, Yb, Lu, Hf, Ta, Th, U, Sc, Cr, Co and CaO. The resulting concentrations were normalized by NASC (North American Shale Composite) which is a good proxy of abundance of rare Earth elements and other lithophiles in sedimentary rocks^41^.

The oxygen and hydrogen isotope compositions were determined in the water samples collected in the study area using the isotope mass-spectrometry methods^42^. The oxygen isotope composition was determined using the CF-IRMS method by *Thermo Delta V* +mass-spectrometer and *GasBenchII* option. Water equilibration was performed at 32 °С during 18 hours. The accuracy of *δ*^18^*O* determination was equal to ±0.05‰. The resulting compositions were normalized by VSMOW – SLAP scale. The hydrogen isotope composition was determined using the chromium reduction method at 800 °С by *Thermo DeltaPlus* mass-spectrometer and *H/Device*. The accuracy of *δD* determination was equal to ±0.3‰.

The atmospheric influence on the Ob and Yenisei plumes was examined using wind data obtained from a 12 h NCEP/NCAR atmospheric reanalysis with a 2.5-degree resolution^43^. The time series of the net surface Ekman transport at the study region for ice-free periods (July–October) were calculated in the following manner. First, the wind stress field ***τ*** was computed from the wind speed field ***v*** according to the equation ***τ*** = *ρ*_*a*_*C*_*D*_***v***|***v***|, where *ρ*_*a*_ is the density of the air, *C*_*D*_ is the drag coefficient prescribed equal to 0.0013^44^. Second, zonal and meridional components of the surface Ekman transport (*M*_*x*_, *M*_*y*_) were calculated as *M*_*x*_ = *τ*_*y*_/*f*, *M*_*y*_ = −*τ*_*x*_/*f*. Finally, the obtained values of *M*_*x*_ and *M*_*y*_ were averaged over the study area (72.5–75 °N, 72.5–80°E) for a period of 3 days. Discharge measurements at the Ob and Yenisei rivers were performed at the most downstream gauge stations situated in the cities of Salekhard and Igarka, respectively.

#### Additive geochemical criterion

The additive geochemical criterion is used to identify origin of a sediment sample if its two or several possible sources are a priori known. These sources are presumed to have different stable geochemical tracers, i.e., the sources can be distinguished based on presence/absence of these tracers. However, concentrations of these trace elements can be relatively low and their determination errors can be of the same order as their absolute values. In this case, a straightforward identification of sediment origin based on determined absolute values of concentrations of individual tracers is prone to large uncertainty. The main advantage of the additive geochemical criterion consists in fact that a sum of normalized concentrations of all trace elements typical for a certain source is more indicative than concentrations of individual elements.

The additive geochemical criterion (*AGC*) for a sediment sample in case of two possible sources is calculated as follows: *AGC* = ln(*E*_1_/*E*_2_), where *E*_1_ = (*X*_1_^1^ + … + *X*_*n*_^1^)/*n*, *E*_2_ = (*X*_1_^2^ + … + *X*_*m*_^2^)/*m* are the sums of normalized concentrations of trace elements in suspended sediments of the first (*X*_1_^1^, …, *X*_*n*_^1^) and second (*X*_1_^1^, …, *X*_*m*_^1^) sources. Sediments from the first source are presumed to have large value of *E*_1_ and small value of *E*_2_, therefore *E*_1_/*E*_2_ > 1, while sediments from the second source result in the opposite inequality, namely, *E*_1_/*E*_2_ < 1. As a result, a sample of bottom sediments is regarded to have origin of the first/second source if the related value of *AGC* is greater/smaller than zero. However, small absolute value of this criterion indicates that a considered sample has mixed origin, i.e., from both sources, and/or presence of large fraction of sediments from different source. The additive geochemical criterion is described in detail in^19^. The main advantage of the additive approach is its high performance at low computational cost. A significant drawback of the approach consists in its instability to fluctuations and outliers, i.e., non-regular anomalous concentration of any trace element result in strong distorting effect to the additive set.

#### Bayesian geochemical criterion

The Bayesian approach is a statistical method based on estimation of probability for a hypothesis by testing this hypothesis using the available information. This approach is widely used to analyze statistical relationships among the available data and combine evidence to predict unknown information in many branches of geophysical science including geology^45–47^, hydrology^48–50^, oceanography^51,52^, atmospheric sciences^53,54^, etc.

Bayesian models of pattern recognition operate with conditional probabilities of attributes, e.g., concentrations of chemical elements as in the current study, and do not directly deal with values of these attributes. Therefore, the Bayesian geochemical criterion does not regard individual chemical elements and compounds as the tracers of suspended sediments of the Ob and Yenisei origin. The mean values of concentrations of these elements and compounds in the set of analyzed samples are relatively small as compared to their dispersions. As a result, the concentrations of individual elements and compounds do not provide enough confident information about the origin of the sediment samples as typical tracers do. However, configurations of their concentrations and statistical relations between them provide significantly more information and can be evident of the origin of sediment samples. In particular, an individual chemical element can have similar mean concentrations, but different ranges in the Ob and Yenisei suspended sediments. The Bayesian geochemical criterion detects these differences in ranges and uses them to distinguish sediments of the Ob and Yenisei origin. Therefore, the Bayesian geochemical criteria provide accurate and stable results in case of anomalous values of attributes and do not depend on their statistical distributions.

In this study we used a multidimensional version of the Bayesian approach adapted to geochemical data, which is commonly used for various geology applications^55^. The general scheme of distinguishing bottom sediments of the Ob and Yenisei origin is the following. We determine a set of chemical elements and compounds *X*_1_, …, *X*_*n*_, which concentrations in samples of bottom sediments are used to detect their origin. Then we consider samples of bottom sediments as points in a *n*-dimensional space, which dimensions are concentrations of *X*_1_, …, *X*_*n*_. Samples of the Ob origin represented by points in this *n*-dimensional space are separated from samples (points) of the Yenisei origin by a (*n* − 1)-dimensional hypersurface. This space and hypersurface can be projected to a 2-dimensional line and a 1-dimesional separating point using a Kullback–Leibler information approach^56^.

We consider two alternative hypotheses, namely, the Ob (hypothesis *H*_*A*_) and Yenisei (hypothesis *H*_*B*_) origin of a sediment sample. Then, based on the determined values of concentrations of *X*_1_, …, *X*_*n*_ in the sediment samples, we define their ranges [*a*_1_, *b*_1_], …, [*a*_*n*_, *b*_*n*_] and divide these ranges into a prescribed number of intervals: $[{a}_{i},{b}_{i}]={\cup }_{k=1}^{m}[{a}_{i}^{k},{a}_{i}^{k+1}]$, where *a*_*i*_^1^ = *a*_*i*_, *a*_*i*_^*m*+1^ = *b*_*i*_. Then for all *i* = 1, …, *n* and *k* = 1, …, *m* we prescribe hypotheses *X*_*i*_^*k*^ that concentration of *X*_*i*_ is within the range [*a*_*i*_^*k*^, *a*_*i*_^*k*+1^]. Then we calculate *P*(*X*_*i*_^*k*^|*H*_*A*_) and *P*(*X*_*i*_^*k*^|*H*_*B*_) that are the conditional probabilities that concentration of *X*_*i*_ is within the range [*a*_*i*_^*k*^, *a*_*i*_^*k*+1^] for a sediment sample of the Ob and Yenisei origin, respectively. Based on these values, we can define if determined concentrations of chemical elements and compounds in sediment sample support hypothesis about the Ob (*H*_*A*_) or Yenisei (*H*_*B*_) origin of this sample in the following way. *P*(*H*_*A*_|*X*_*i*_^*k*^) and *P*(*H*_*B*_|*X*_*i*_^*k*^) are the probabilities of hypotheses that the considered sediment sample has the Ob or Yenisei origin if the determined concentration of *X*_*i*_ in this sample is within the range [*a*_*i*_^*k*^, *a*_*i*_^*k*+1^]. According to Bayes’ law *P*(*H*_*A*_|*X*_*i*_^*k*^) = *P*(*X*_*i*_^*k*^|*H*_*A*_) ∙ *P*(*H*_*A*_)/*P*(*X*_*i*_^*k*^), *P*(*H*_*B*_|*X*_*i*_^*k*^) = *P*(*X*_*i*_^*k*^|*H*_*B*_) ∙ *P*(*H*_*B*_)/*P*(*X*_*i*_^*k*^), where *P*(*X*_*i*_^*k*^) is the probability that concentration of *X*_*i*_ is within the range [*a*_*i*_^*k*^, *a*_*i*_^*k*+1^] for all sediment sample, i.e., samples of any (Ob, Yenisei, or mixed) origin, *P*(*H*_*A*_) and *P*(*H*_*B*_) are the probabilities that a sediment sample is of the Ob and Yenisei origin, respectively. Therefore, we calculate the ratio of probabilities of two hypothesis, i.e., likelihood ratio, *P*_*i*_ = *P*(*H*_*A*_|*X*_*i*_^*k*^)/*P*(*H*_*B*_|*X*_*i*_^*k*^) = *P*(*X*_*i*_^*k*^|*H*_*A*_) ∙ *P*(*H*_*A*_)/*P*(*X*_*i*_^*k*^|*H*_*B*_) ∙ *P*(*H*_*B*_), logarithm of this ratio provides an informational measure similar to the Kullback information. The sum of the amount of this information from all considered elements and compounds is value of the Bayesian geochemical criterion $BG{C}_{O/Y}={\sum }_{i=1}^{n}ln({P}_{i})$ used for recognition of bottom sediments of the Ob (positive values) and Yenisei (negative values) origin.

## Supplementary information

Dataset 1

Dataset 2

## Data Availability

The NCEP/NCAR reanalysis data were downloaded from the National Oceanic and Atmospheric Administration website https://www.esrl.noaa.gov/psd/data/. The river discharge data were received from the Global Runoff Data Centre, 56068 Koblenz, Germany. The *in situ* data are available in supplementary information.
